# Biogenic gold nanoparticles *via Aloe vera* waste: a sustainable catalyst for water treatment

**DOI:** 10.1039/d6ra09195d

**Published:** 2026-10-28

**Authors:** Lucia Mergola, Agostina Lina Capodilupo, Antonio Serra, Daniela Manno, Alessandro Buccolieri, Roberta Del Sole

**Affiliations:** a Department of Engineering for Innovation, University of Salento, Campus Ecotekne Via per Monteroni 73100 Lecce Italy lucia.mergola@unisalento.it roberta.delsole@unisalento.it; b Institute of Nanotechnology (CNR-NANOTEC), Campus Ecotekne Via per Monteroni 73100 Lecce Italy; c Department of Mathematics and Physics “E. De Giorgi”, Campus Ecotekne Via per Monteroni 73100 Lecce Italy

## Abstract

Starting from the serious environmental problems associated with the growing increase of pollution, the present study investigates a sustainable and eco-friendly approach to water treatment by synthesizing green gold nanoparticles (AuNPs) starting from *Aloe barbadensis* Miller (*Aloe vera*) waste (AVW). Bioactive compounds naturally present in AVW were extracted in water at different temperatures (25–90 °C). The extract prepared at 90 °C was identified as the most suitable for stable AuNPs formation, containing higher amounts of reducing and stabilizing agents such as α- and β-glucose, sucrose and malic acid, as confirmed by ^1^H-NMR studies. The appearance of a typical plasmonic band at 560 nm confirmed the success of AuNPs synthesis. Selected-area electron diffraction and transmission electron microscopy analyses demonstrated the crystalline nature of the synthesized AuNPs with an average diameter of 10 nm and with a zeta potential of −32.3 mV. Moreover, FTIR analysis revealed the presence of an organic layer around the AuNPs, also confirmed from UV-vis analysis. Methylene blue (MB), rhodamine b (ROb), methyl orange (MO) and 4-nitrophenol (4NP) were selected as water pollutant models to test their catalytic activity. The results obtained showed the high capacity of green AuNPs to speed up the reduction rate following a pseudo first order kinetic with *k* values observed for MB, ROb, MO and 4NP of 0.32, 0.15, 0.09 and 0.04 min^−1^ respectively. This behavior remains highly efficient even when all pollutants are present simultaneously in a mixture, demonstrating their potential use also in complex matrices.

## Abstract 2

*Aloe vera* waste valorization through the synthesis of green gold nanoparticles applied in water remediation from thiazine-dyes, xanthene-dyes, azo-dyes, and nitroarenes.

## Graphical abstract

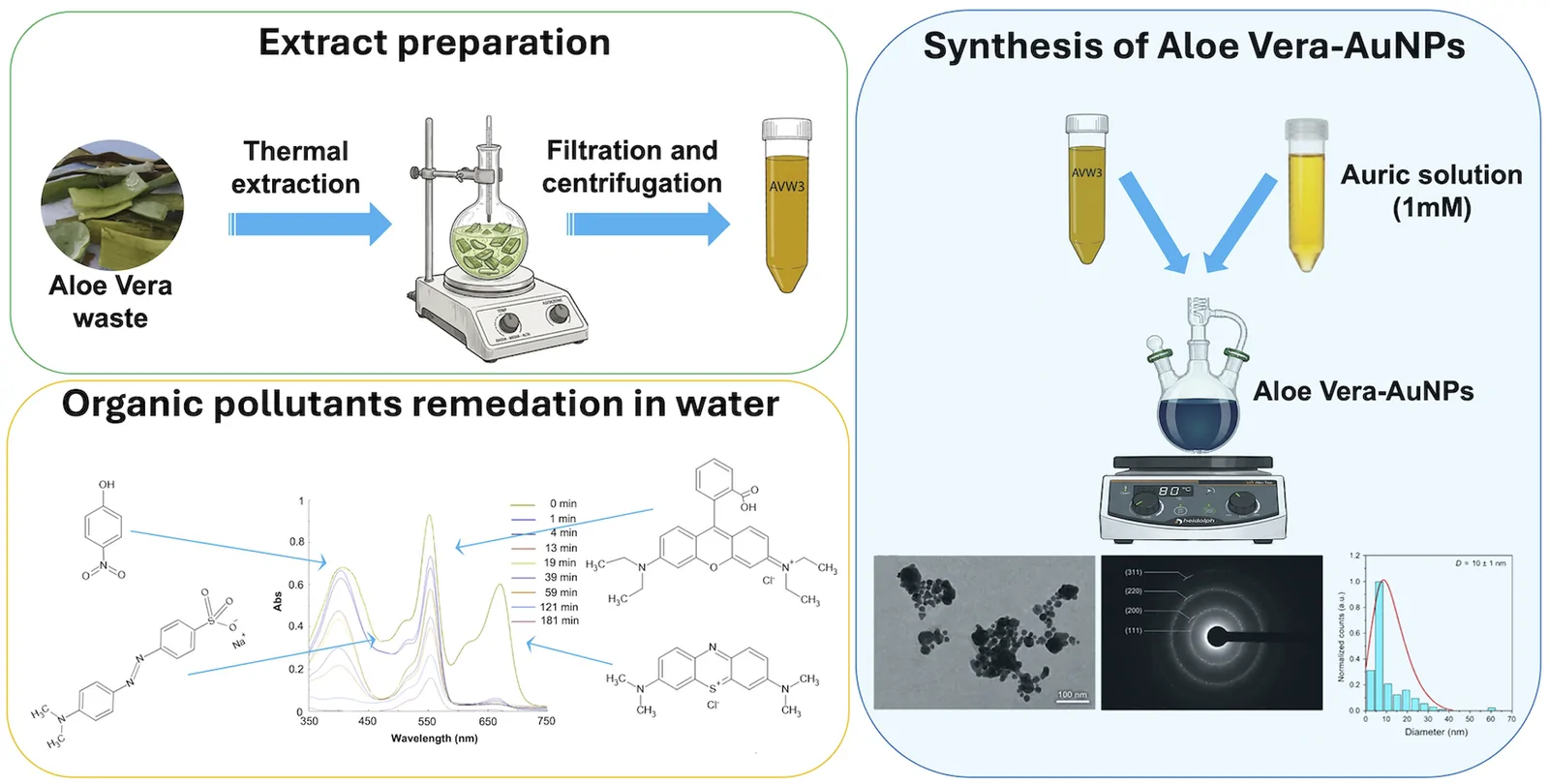

## Introduction

Water pollution is a critical global problem that, in the last century, with the increase of industrialization, has become particularly relevant, due to severe, life-threatening dangers to both human health and fragile aquatic ecosystems worldwide.^1^ Industrial discharges release in the environment high concentrations of pesticides, solvents and organic pollutants strongly associated with the increase of cancer diseases and endocrine disruption. Thiazine-dyes, xanthene-dyes and azo-dyes, together with nitroarenes, represent the major categories of contaminants generally found in industrial wastewater due to their extensive use as raw materials and chemical intermediates in manufacturing and to their high solubility and persistence in water.^2,3^ The presence in their structures of complex aromatic rings confer to these compounds natural stability and high resistance to traditional water treatment, amplifying their long-term threats. Their persistence in the environment represents the key element of their toxicity not only for human beings but also for aquatic plants and animals. Intensive agricultural treatments and the discharge of wastewater from textile, pharmaceutical and printing industries represent the principal pollution sources of these compounds^3,4^ This evidence justifies the high interest of the scientific community in finding new eco-friendly and sustainable methodologies to eliminate these compounds or to reduce their toxicity. Common methodologies generally applied to wastewater treatments consist of membrane filtration, adsorption, coagulation and flocculation, sedimentation, filtration and disinfection.^5^ However the use of these methodologies often causes the production of hazardous by-products which can be more toxic than the starting substances. For these reasons, the research of new strategies to drastically reduce their toxicity represents an important goal. Starting from the concept of “green nano” introduced for the first time from Anastas,^6^ in recent years, a lot of studies have been focused on the development of green and sustainable nanotools applied in water remediation, prepared replacing common hazardous reagents with natural substances obtained from plants, fruits, agriculture waste and microbes.^7–9^ Preparation of noble metal nanoparticles starting from natural products, represents today an interesting research topic for the sustainability of the preparation process but also to the unique optical, electronic and catalytic properties of these nanotools which allow its use in a wide range of application fields. Aqueous vegetable extracts, naturally rich in sugars, organic acids and polyphenols, can act as natural reducing and stabilizing agents in noble metal nanoparticles preparation, avoiding the use of hazardous compounds that could worsen environmental pollution and limit their application in medical field.^7,10–13^ Often, the use of green methodology leads to the formation of a typical organic layer around noble metal nanoparticles which contributes to increasing their catalytic efficiency compared to conventional catalysts.^14^ In the last years, the preparation of green gold nanoparticles (AuNPs) applied to catalytic water remediation has been gaining a lot of attention due to their high stability and low toxicity with high catalytic performance that, associated with their massive surface area, enables rapid contaminants removal.^15^ A lot of vegetable extracts were used in AuNPs preparation.^11,12,16,17^ Agricultural waste such as food processing by-products, crop waste and plant residues, have been receiving much attention due to their interesting composition associated with the high presence of bioactive compounds.^18–21^ In the present study *Aloe vera Barbadensis* Miller waste (AVW), obtained from a local cosmetic manufacturing company (Natura & Benessere s.r.l.) after mechanical removal of the gel, was processed to obtain starting material for AuNPs synthesis. This perennial plant, rich in important bioactive compounds, is renowned for its healing properties and widely used in the treatment of skin lesions and digestive problems.^22^ The high demand of gel-based products causes the production of high amounts of AVW that can be transformed into value-added products such as fertilizers, biofuels, animal feeds and biosorbents. Indeed, chemical characterization of AVW show the presence of important substances such as amino acids, monosaccharides, polysaccharides, vitamins, organic acids, lipids, minerals, phenolic compounds that justify all efforts for its valorization, also in view of a circular bioeconomy management.^23^ To this aim, AVW can be considered as an undisputed source of natural reducing and stabilizing agents in AuNPs preparation. In the last years, starting from the well-known anti-inflammatory and antimicrobial properties, aqueous *Aloe vera* extracts were used to prepare green AuNPs mainly applied in microbial disinfection.^24–28^ In a recent work a blue-green emitting gold nanoclusters were prepared using *Aloe Africana* leaf juice extract and their cytotoxicity and antimicrobial properties against *Staphylococcus Aureus* and *Escherichia coli* were demonstrated.^25^ In another work Néel and co-workers described the functionalization of *Aloe vera*-AuNPs with 16-mercaptohexadecanoic acid or l-cysteine obtaining nanoparticles and nanoplatelets further functionalized to enhance Raman signal, demonstrating their potential use in SERS applications.^29^ To date, no relevant works have been conducted on the use of *Aloe vera*-AuNPs in catalytic water remediation. Starting from the negative impact for human health and for the environment demonstrated from the high presence in wastewater of dyes and nitroarenes, the present work focused its attention on the development of an eco-friendly and sustainable nanotool prepared starting from AVW extracts and its application in water detoxification. After one-step preparation and an extensive characterization, green AuNPs were tested in catalytic reduction of methylene blue (MB) and rhodamine b (ROb) chosen as cationic dye models, methyl orange (MO) anionic dye model, and 4-nitrophenol as nitroarene model in order to turn the above pollutants in less harmful compounds. Finally, their catalytic activity was also evaluated on a mixture solution containing simultaneously all tested compounds to simulate more complex matrices.

## Experimental

### Materials

AVW was kindly supplied from a local cosmetic manufacturing company (Natura & Benessere s.r.l.), specialized in the preparation of *Aloe vera*-based cosmetic formulation, starting from *Aloe barbadensis* Miller plants (*Aloe vera*), cultivated in the southern of Apulia. Chloroauric acid (HAuCl_4_·3H_2_O, 99.99%), deuterated water (D_2_O), gallic acid (GA), MB, MO, 4-NP, ROb, sodium carbonate (Na_2_CO_3_, 99%), copper(ii)sulfate pentahydrate (CuSO_4_), sodium hydroxide (NaOH), potassium sodium tartrate and sodium borohydride (NaBH_4_), were purchased from Sigma-Aldrich (Steinheim, Germany). Folin–Ciocalteu's phenol reagent was supplied from Merck KGaA (Darmstadt, Germany). Ultrapure water, used in the preparation of all solutions, was produced by a water purification system (Human Corporation, Seoul, Korea).

### *Aloe vera* waste treatment

AVWs, which include part of leaves and gel remaining after the gel extraction production process, was cut into small pieces of about 1 cm and subjected to extraction with 150 mL of ultrapure water for 1 hour. Different extraction conditions were used as described in Fig. 1. Then, AVW extracts were coarsely filtered using a vacuum pump and further purified by centrifugation at 6000 rpm for 10 minutes by using a PK121 multispeed of Thermo Electron Corporation (Thermo Electron Corporation, Waltham, MA, USA). Each supernatant was aliquoted and frozen at −20 °C until use. AVW extracts pH was also evaluated using a pH meter basic 20, (Crison, Alella, Barcelona, Spain, and Europe).

**Fig. 1:**
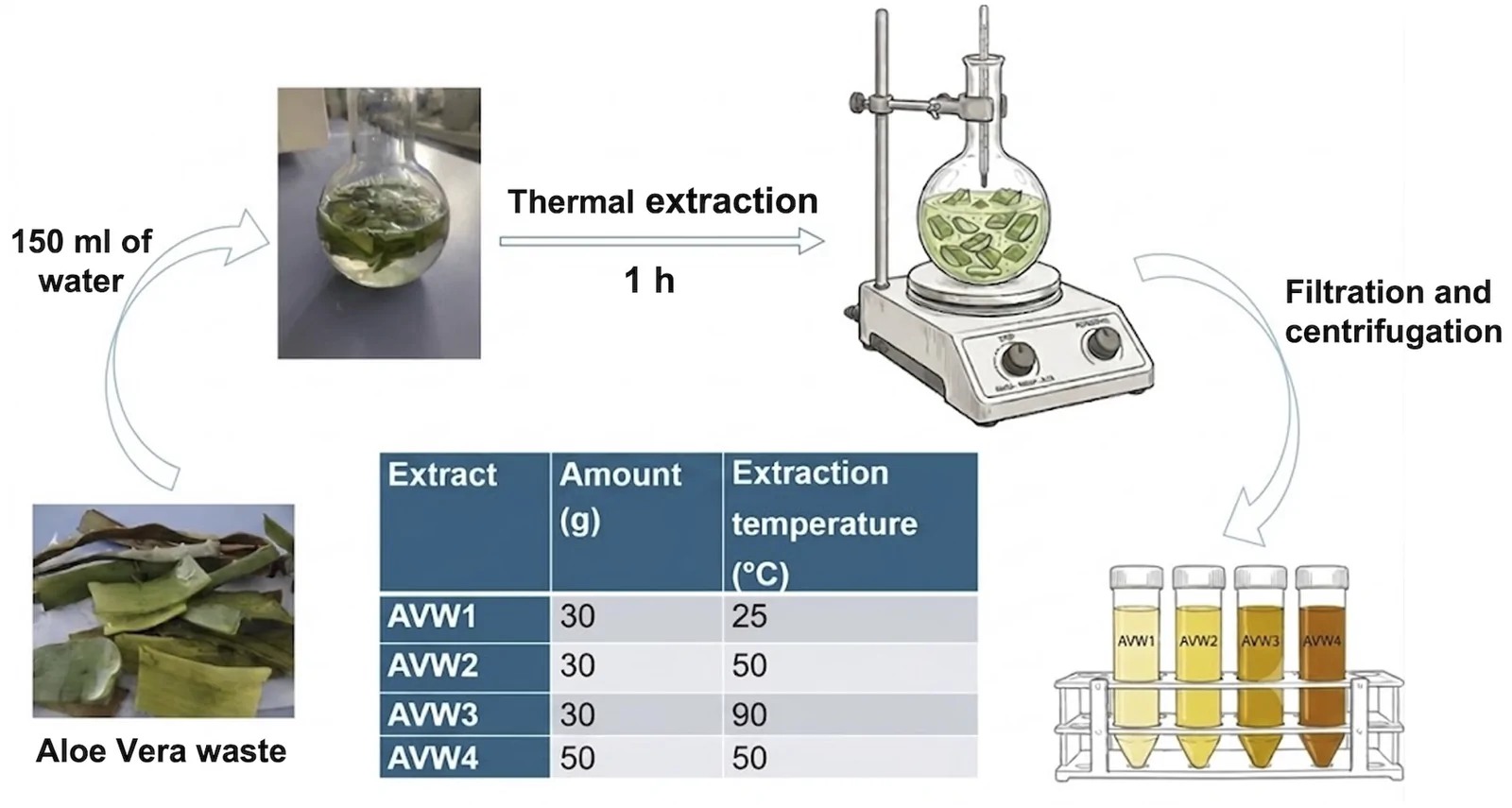
Schematic representation of AVW extracts preparation.

### Green gold nanoparticles preparation

All obtained extracts were used as starting materials in AuNPs preparation. The synthesis was carried out in a three-neck flask equipped with a refrigerant system, adding 5 mL of extract to an auric solution (1 mM), in a final volume of 50 mL. The synthesis was carried out at 80 °C for 1 hour under magnetic stirring (700 rpm) and it was monitored through UV-vis analysis by using a Jasco spectrophotometer (V-660 UV-vis, Jasco, Palo Alto, CA, USA). Then, all samples were centrifuged at 8000 rpm for 10 minutes and resuspended in ultrapure water. After a total of 7 centrifugations, all purified samples were lyophilized and stored at room temperature until use.

## Characterization studies

### Total phenolic content (TPC) of AVW extracts

TPC was evaluated by a spectrophotometric Folin–Ciocalteu modified method^30^ using gallic acid (GA) as standard for calibration curve preparation in a range of 0–350 mg L^−1^. Briefly, 125 µL of each not diluted extract or GA standard solution was added to 0.5 mL of ultrapure water in a test tube. Then, 125 µL of Folin–Ciocalteu reagent was also added and the reaction mixture was vigorously mixed and allowed to react for 6 min in the dark before the neutralization with 1.25 mL of a 7% sodium carbonate aqueous solution. A final volume of 3 mL was reached using ultrapure water. The reaction was conducted under stirring (200 rpm) for 120 minutes, in the dark, at room temperature. A blank sample was also prepared using only ultrapure water instead of the extract. The absorbance of each solution was evaluated at 760 nm *versus* the blank and compared with known gallic acid concentrations. All experiments were conducted in triplicate, and the results were expressed as mean of gallic acid equivalents (GAE) per gram of Fresh Matter (FM) ± SD.

### Reducing sugars evaluation of AVW extracts

Reducing sugars content was evaluated using a Fehling method. Firstly, Fehling's Solution A (deep blue copper sulfate) and Fehling's Solution B (clear sodium hydroxide and potassium sodium tartrate) were prepared and mixed in equal volumes. After the addition of 2 drops of MB solution (1%) used as indicator, the mixture was heated and, after boiling, it was titrated with the AVW extract until the formation of a red precipitate and a color change from blue to colorless confirmed the complete reduction of the Fehling solution. The extract volume used for each analysis was adopted to quantify the reducing sugars present in each extract using the following equation:

1 [Monosaccarides] (g L^−1^) = 0.0515 × 1000 × *D*/*A*

where *D* and *A* represent the dilution factor and the AVW extract volume (mL) used for titration respectively.^11^

### AVW-AuNPs characterization studies

Diluted colloidal suspensions of AVW-based gold nanoparticles (AuNPs@AVW) were firstly characterized by UV-vis spectrophotometer to confirm nanoparticles formation from the appearance of a specific plasmonic band. Zeta-potential was also evaluated on diluted samples using Malvern Zetasizer nano ZS 90 (Worcestershire, UK) to assess the stability of the NPs colloidal suspension and the charge of the external shell. Fourier transform infrared spectroscopy (FTIR) analyses were conducted using a JASCO 660 plus infrared spectrometer (Palo Alto, CA, USA) in a range of 4000 and 650 cm^−1^. A Rigaku MiniFlex diffractometer equipped with CuKα radiation (*λ* = 1.5418 Å) was used to perform X-ray diffraction studies, operating at 30 kV and 15 mA. All data were acquired in the 2*θ* range of 20–80°, with a step size of 0.02° and a scan rate of 2° min^−1^. Transmission electron microscopy (TEM) analyses were performed using a Hitachi H-7700 microscope with an accelerating voltage of 120 kV. Diluted colloidal suspensions were drop-casted onto a carbon-coated copper grid and then dried at room temperature. To minimize electron-beam-induced morphological alterations or nanoparticle sintering, observations were conducted using minimal exposure times and low current densities. High-resolution transmission electron microscopy (HRTEM) images of representative areas were acquired to determine nanoparticle shape and size, complemented by selected-area electron diffraction (SAED) patterns to assess their crystallinity. NMR measurements were carried out on a Bruker Avance NEO 400 MHz spectrometer. Freeze-dried AVWs were dissolved in D_2_O and ^1^H NMR spectra were recorded using the standard one-dimensional zg30 pulse sequence (Bruker). AVW extracts obtained at 50 °C and 90 °C were analyzed by ^1^H NMR spectroscopy. The principal metabolites naturally present in whole-leaf *Aloe vera* were identified based on their characteristic ^1^H NMR resonances.

### Catalytic evaluation and kinetic studies of AVW-AuNPs

Catalytic performance of AuNPs@AVW were evaluated on the reduction of different organic pollutants in water in the presence of NaBH_4_ used as a reducing agent. In detail, three toxic colorants (MB, MO and ROb) and a phenolic pollutant (4NP) were tested. 9 mL of pollutant solution (42 mg L^−1^) kept under nitrogen flow for 1 minute, was mixed with 1 mL of NaBH_4_ solution (100 mM) and stirred for three minutes. Then, 0.5 mL of AuNPs@AVW (144 µg mL^−1^) or ultrapure water were added to the mixture reaching a catalyst dosage of 7 µg mL^−1^ in the final volume. After 20 seconds, the UV-vis spectra were recorded at regular intervals of time. Each pollutant was monitored at different wavelengths (400 nm for 4NP, 464 nm for MO, 554 nm for ROb and 664 nm for MB) and the absorbance at the time *t* (*A*_*t*_) was recorded. The % of pollutant reduction was calculated as follows:

2 Reduction (%) = (1 − *A*_*t*_/*A*_0_) × 100

where *A*_*t*_ and *A*_0_ represent the absorbance at the time *t* and 0, respectively. All catalytic experiments were conducted in triplicate. Moreover, kinetic studies were performed by using both pseudo first order and pseudo second order kinetic models as follows:

3 LN(*A*_*t*_/*A*_0_) = −*kt*

4 1/*A*_*t*_ − 1/*A*_0_ = *kt*

Finally, further two set control studies were performed for all pollutants. The same catalytic procedure above described was used with the following differences. The first control study was conducted in the absence of NaBH_4_ (replaced with ultrapure water). While the second one was conducted in the absence of NaBH_4_ and AuNPs@AVW, replaced with 1 mL of ultrapure water and 0.5 mL of diluted extract respectively.

## Results and discussion

### AVW extract preparation and characterization

AVW, kindly provided from a local cosmetic manufacturing company, consists in leaves pieces of different sizes and *Aloe vera* gel residues containing important water soluble bioactive compounds that, if extracted, can be used as starting materials in gold nanoparticles preparation instead of toxic substances commonly used in synthetic processes. Polysaccharides, organic acids, vitamins and enzymes represent the main components of the gel, associated with minerals, phenolic compounds, flavonoids and anthraquinones found in the leaves.^23^ The presence of carbonylic and hydroxylic groups in their structures makes these substances potential reducing and stabilizing agents, useful in green nanoparticles preparation.^14^

Although organic solvents such as methanol,^31^ ethanol or their mixture with water^32,33^ were usually used in the extraction of bioactive compounds from *Aloe vera*; in this work, ultrapure water as green extraction solvent was preferred since, thanks to its high polarity, favors the extraction of phenolic compounds and reducing sugars as well, essential to maximize the reducing power of the extracts and at the same time, to ensure environmental sustainability, a crucial thread of the present work. Some different experimental conditions were considered in the extraction process (Fig. 1). To evaluate the influence of temperature in the extract composition and the suitability of the extract as starting material in nanoparticles formation, different extraction temperatures ranging from 25 to 90 °C were used and TPC and reducing sugars (RS) of the prepared extracts were calculated. The results obtained showed a direct correlation between temperature, total phenolics and RS content. As can be seen in Table 1, enhancing the extraction temperature (25 °C, 50 °C and 90 °C for AVW1, AVW2 and AVW3 respectively), both total phenolic and RS content increase. A second attempt, conducted keeping the temperature constant at 50 °C and enhancing the amount of waste from 30 g (AVW2) to 50 g (AVW4), showed a small increase of these parameters. The obtained results highlight the crucial role of temperatures in extract composition, in line with some different literature evidence.^11,34,35^ Although the extraction process was conducted using only ultrapure water, the results obtained in terms of TPC are comparable to the results described in other works that use organic solvents such as methanol and ethanol.^23^

**Table 1:** Characterization of AVW extracts in terms of TPC, reducing sugars and pH

AVW extract	pH	TPC (gGAE/100g FM)	RS (g L^−1^)	Yield (g/100g FM)
AVW1	4.7	0.32 ± 0.02	2.7	—
AVW2	4.6	0.53 ± 0.02	3.5	—
AVW3	4.7	1.23 ± 0.03	11.4	3.4
AVW4	4.6	0.72 ± 0.02	5.6	0.6

These results can be explained by the distinctive agro-climatic characteristics of the Mediterranean area, with intense solar radiation, heat, and drought which probably lead to an increase in phenolic content.^36^ and drought which probably lead to an increase in phenolic content.^36^ Finally, AVW3 and AVW4, which showed the best results, were analyzed by ^1^H NMR spectroscopy (Fig. 2). The principal metabolites naturally present in whole-leaf *Aloe vera* were identified based on their characteristic ^1^H NMR resonances. The spectra revealed the presence of the major carbohydrate components, including sucrose, α- and β-glucose, and acemannan, together with low-molecular-weight organic acids such as malic acid, lactic acid and acetate (Fig. 3). The assignments were based on the characteristic chemical shifts, multiplicities, and coupling constants reported for these metabolites. Representative ^1^H NMR spectra of the extracts are shown in Fig. 2. In detail, as can be seen in Fig. 2, the ^1^H NMR spectrum of the *Aloe vera* extract showed several characteristic resonances attributable to the main marker components of the plant matrix. Sucrose was identified by the anomeric proton of the glucose unit at 5.41 ppm (d, *J* = 3.8 Hz), together with resonances of the fructose unit at 4.20 ppm (d, *J* = 8.8 Hz) and 4.04 ppm (t, *J* = 8.8 Hz). The anomeric protons of free α- and β-glucose were observed at 5.22 ppm (d, *J* = 3.8 Hz) and 4.63 ppm (d, *J* = 8.0 Hz), respectively. A doublet at 5.05 ppm was attributed to the anomeric proton of a β-linked sugar residue, most likely associated with mannosyl units of deacetylated acemannan or acemannan-derived oligosaccharides in accordance with the very low signals of the acetyl protons typically present at around 2.0–2.2 ppm probably due to the deacetylation process favored from the high temperature used in the extraction process (90 °C) of acemannan. Moreover, a crowded region of sugars skeleton (glucose, and fructose and mannosyl units) at 3.2–3.90 ppm was observed. Malic acid was confirmed by its characteristic methine resonance at approximately 4.3–4.4 ppm and by the two diastereotopic methylene proton signals between 2.6 and 2.8 ppm. In addition, lactic acid was identified by the coupled resonances of the methine proton at approximately 4.10 ppm and the methyl group at about 1.28 ppm. Minor organic acids were also detected, including pyruvic acid, assigned to the singlet at approximately 2.36 ppm, while signals in the 2.0–2.2 ppm region were consistent with acetate and/or the *O*-acetyl groups of acemannan. Even if all the above defined signals were detected in both spectra (Fig. 2A and B) the ratio of them are significantly different. A high content of free α- and β-glucose was found in the extract AVW4 (Fig. 2A) while increasing the extraction temperature in addition to the glucose, other organic molecules were also extracted, in fact in AVW3 (Fig. 2B) sucrose, and malic acid are the main extract compounds. Moreover, a higher yield was obtained for AVW3 (3.4 g/100 g of FM) compared to the extract obtained at 50 °C (0.58 g/100 g of FM).

**Fig. 2:**
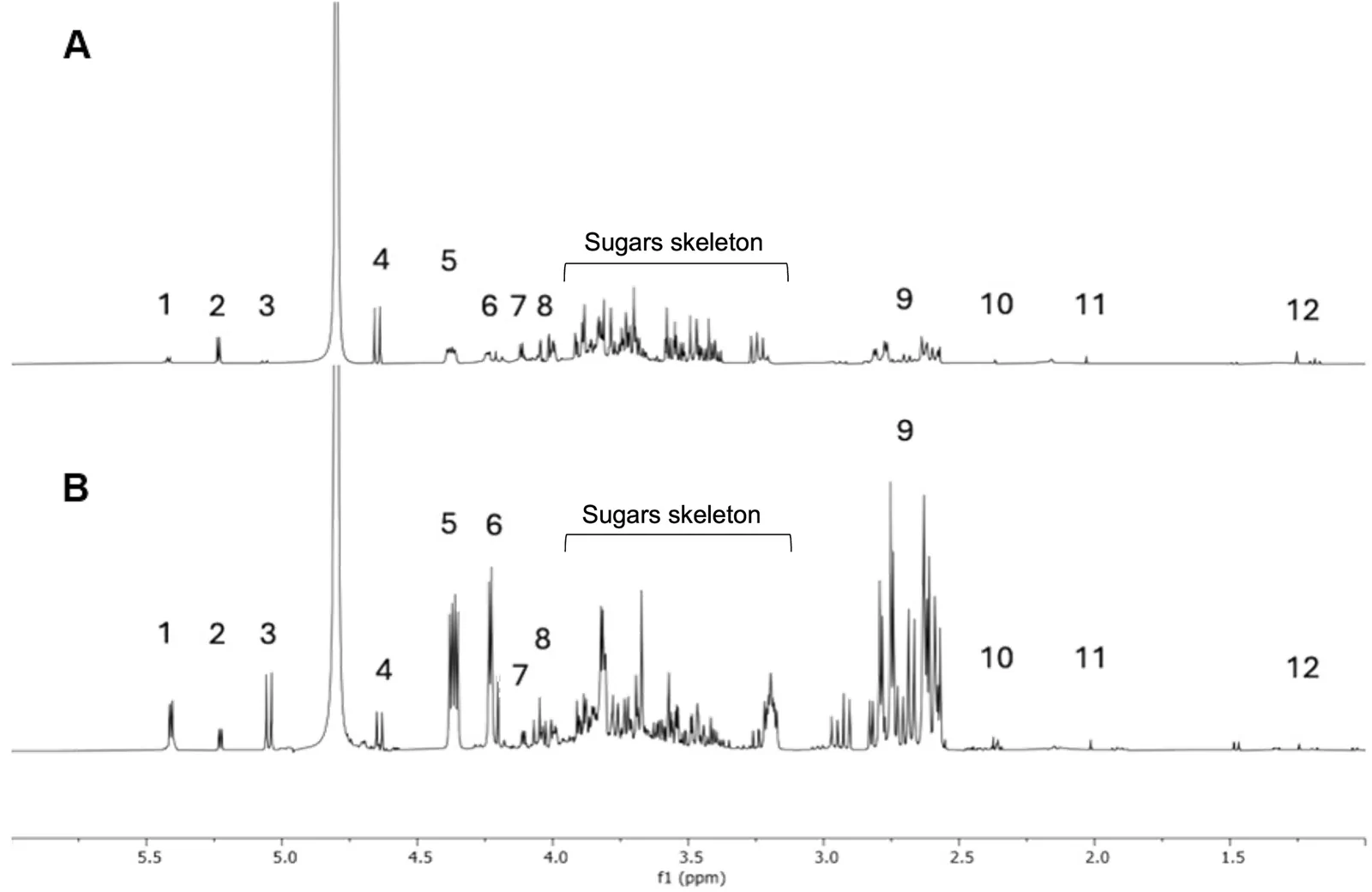
1H NMR spectra of AVW4 (A) and AVW3 (B). Peak assignments: (1) H-1 of the glucose unit of sucrose; (2) anomeric proton of free α-glucose; (3) anomeric proton of a β-linked sugar residue (most likely associated with acemannan); (4) anomeric proton of free β-glucose; (5) H-2 of malic acid; (6) H-3 of the fructose unit of sucrose; (7) methine proton (CH) of lactic acid; (8) H-4 of the fructose unit of sucrose; (9) H-3a/H-3b methylene protons of malic acid; (10) methyl group of pyruvic acid; (11) acetate and/or *O*-acetyl methyl groups of acemannan; (12) methyl group of lactic acid.

### Synthesis and characterization of green gold nanoparticles

Although the presence of reducing sugars and high amount of organic acids, combined with higher phenolic content (Table 1), makes AVW3 more suitable in nanoparticles formation, all prepared extracts were anyway tested as starting materials in nanoparticles preparation.

**Fig. 3:**
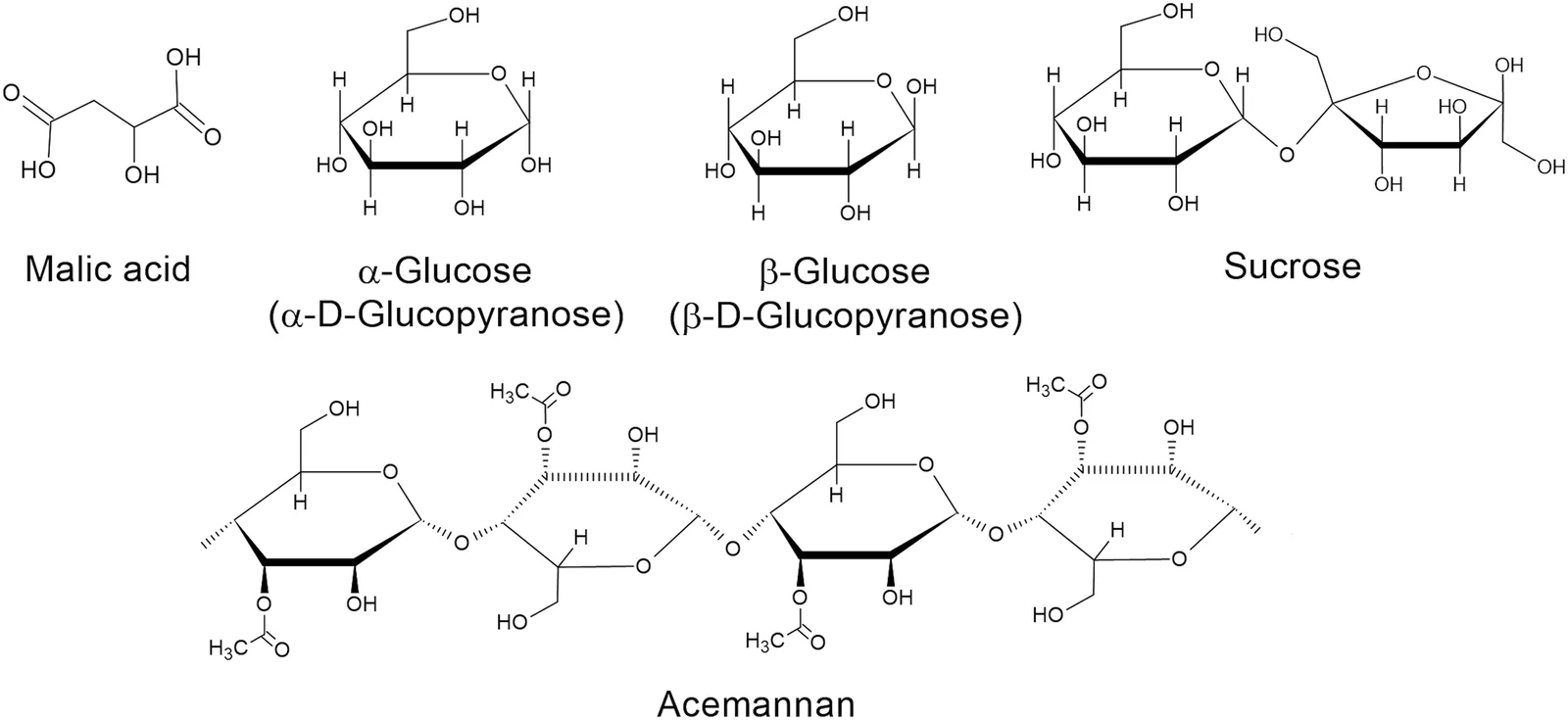
Chemical structures of principal constituents of AVW extracts.

Syntheses performed using AVW1 and AVW2 extracts resulted in the formation of a gold coating on flask walls, probably due to the presence of low amount of stabilizing agents that led to heterogenous nucleation with consequent aggregation and deposition on glass surfaces, as confirmed from the experimental results obtained from extracts characterization (Table 1). Colloidal gold nanoparticles, AuNPs@AVW3 and AuNPs@AVW4, were instead obtained by using respectively AVW3 and AVW4 extracts. However, since AuNPs@AVW4 showed low stability (it can be seen the formation of a precipitate in few hours), AuNPs@AVW3 was selected and subjected to further studies. After purification, colloidal solution of AuNPs@AVW3 was lyophilized (80% yield), resuspended in ultrapure water and characterized. UV-vis studies (Fig. 4A) showed the appearance of a specific plasmonic band at 560 nm that confirmed nanoparticles formation.

**Fig. 4:**
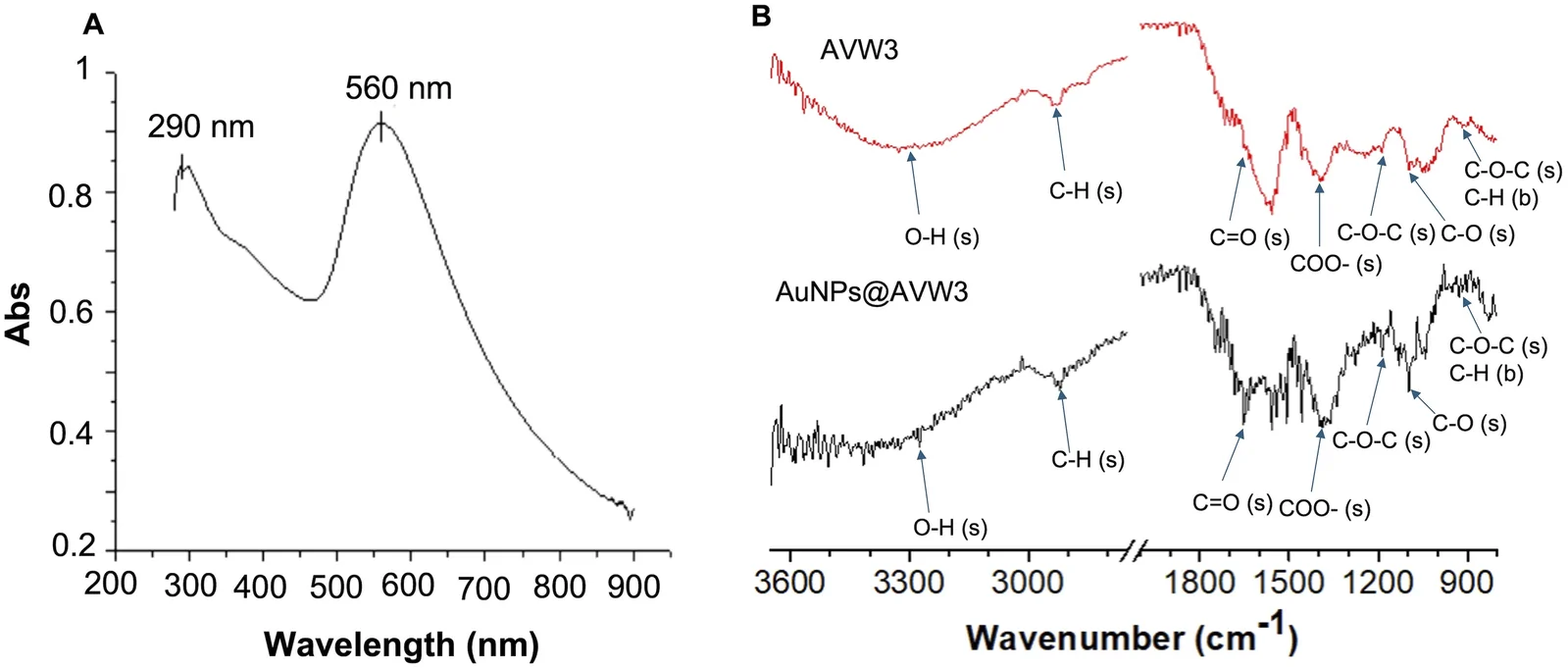
UV-visible spectrum (A) and FTIR (B) of AVW3 and AuNPs@AVW3.

Moreover, the presence of a second peak around 290 nm emphasized the presence of an organic layer around gold nanoparticles that stabilize the colloidal suspension,^13,37^ also supported by FTIR studies (Fig. 4B). Indeed, FTIR spectrum of AVW3 shows various broad signals that confirm the complexity of the extract with the presence of specific peaks associated with sugars (α- and β-glucose and sucrose), polysaccharides (Acemannan) and organic acids (mainly malic acid) (Fig. 4B and Table 2). Each assignment was described in Table 2 in accordance with ^1^H NMR identification previously described. Comparing FTIR spectra of AVW3 and AunPs@AVW3 the expected presence of an organic matter around gold nanoparticles was confirmed by the specific peaks in AuNPs@AVW3 associated to the principal functional groups of sugars and organic acids. Sugars, malic acid and acemannan (Fig. 3) synergically act as reducing and stabilizing agents promoting nanoparticles formation. Moreover, these compounds and their oxidized forms can directly interact with the gold surface through their hydroxyl and carboxyl groups, acting as steric stabilizers and creating a negative organic layer around nanoparticles that enhance electrostatic repulsion, avoiding their aggregation. This data was confirmed from a zeta potential value of −32.3 ± 4.06 mV, typical of gold nanoparticles with excellent stability along with an external layer negatively charged. These results underline the suitability of this extract as alternative green tools in gold nanoparticles formation, instead of common toxic chemical compounds that can drastically limit their use.^38^

**Table 2:** Peak assignments to FTIR spectra of AVW3

AVW3 peak position (cm^−1^)	Assigned functional group/vibrational mode	Biomolecule attribution	Observation
3327	*ν*(O–H) stretching (broad, hydrogen-bonded)	Glucose/mannose/sucrose/malic acid	Hydroxyl matrix
2938, 2905 × 10^2856^	*ν*_as_(C–H), *ν*_s_(C–H) aliphatic stretching	Carbohydrate core/malate backbone	Aliphatic core
1734, 1715, 1698 × 10^1683^	*ν*(C=O) carbonyl stretching (free –COOH)	Malic acid	Carboxylic acid
1456, 1418, 1395	*ν*_s_(COO^−^) symmetric carboxylate stretching	Malic acid (deprotonated fraction)	Carboxylate salt
1189	*ν*(C–O–C) glycosidic link/ring stretching	Monosaccharides & sucrose	Pyranose ring stretch
1098, 1082	*ν*(C–O) carbinol stretching	Malic acid	Secondary alcohol
1053	*ν*(C–O) C–O stretching shoulder	Sucrose component	Sucrose skeletal

AuNPs@AVW3 were also characterized by TEM and crystallographic analyses. As can be seen in Fig. 5A, triangular and spherical individual particles are recognizable, but their closer packing and partial overlap make the core–shell morphology less clear. However, a weak low-contrast halo can be locally detected around some particles. Their crystalline nature was confirmed from the diffraction ring obtained (Fig. 5B) that can be indexed to the face-centered cubic (fcc) structure of metallic gold, with reflections assigned to the (111), (200), (220), and (311) crystallographic planes. The spotty character of the rings indicates that diffraction arises from a polycrystalline ensemble of well-crystallized Au nanodomains with different orientations. Since the coating shell is amorphous, no distinct diffraction contribution is expected from this external layer; therefore, the SAED (Selected Area Electron Diffraction) pattern mainly provides structural information on the crystalline Au cores observed in TEM analysis. Particle-size distribution of AuNPs@AVW3 reported in Fig. 5C, is dominated by a single main population centered at approximately *D* = 10 ± 1 nm. The distribution is shifted toward an intermediate average diameter and shows a pronounced tail toward larger sizes, extending up to several tens of nanometers. This behavior is in agreement with the TEM image, where the presence of same aggregates, overlapping particles and large clusters contribute to an apparent broadening of the measured size distribution. Although ^1^H NMR analysis of gold nanocatalyst could provide important information about the organic layer around gold nanoparticles, unfortunately in our case the nanoparticles of 10 nm showed a strong localized surface plasmon resonance with the paramagnetic influence of metal core that suppress NMR signals.^39^ In fact it is well known that this technique can be used only in the presence of ultrasmall nanoparticles (1–3 nm).

**Fig. 5:**
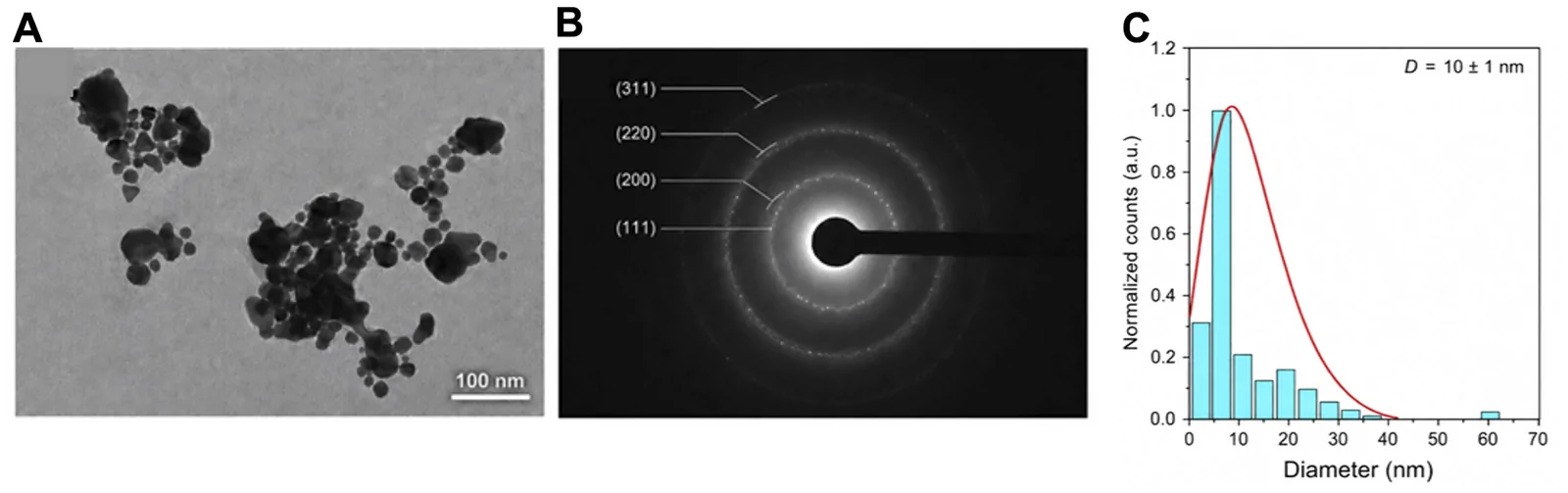
TEM analysis (A), cristallographic studies (B) and particle-size distribution (C) of AuNPs@AVW3.

### Evaluation of catalytic activity of AuNPs@AVW3

To evaluate the catalytic efficiency of the synthesized green gold nanoparticles, cationic and anionic water pollutants were selected.

This study was focused on different dyes-based pollutants and phenolic compounds that represent the principal contaminants found in industrial wastewater. In detail two cationic dyes (MB and ROb), one anionic dye (MO), and a phenolic compound (4NP) were selected. Fig. 6, 7, 8 and 9 show UV-vis spectra, reduction performances and kinetic studies conducted on MB, ROb, MO, and 4NP respectively, comparing for each pollutant, the reduction performance with and without AuNPs@AVW3. All reactions were performed at room temperature and monitored at regular intervals of time by UV-vis spectrophotometer.

**Fig. 6:**
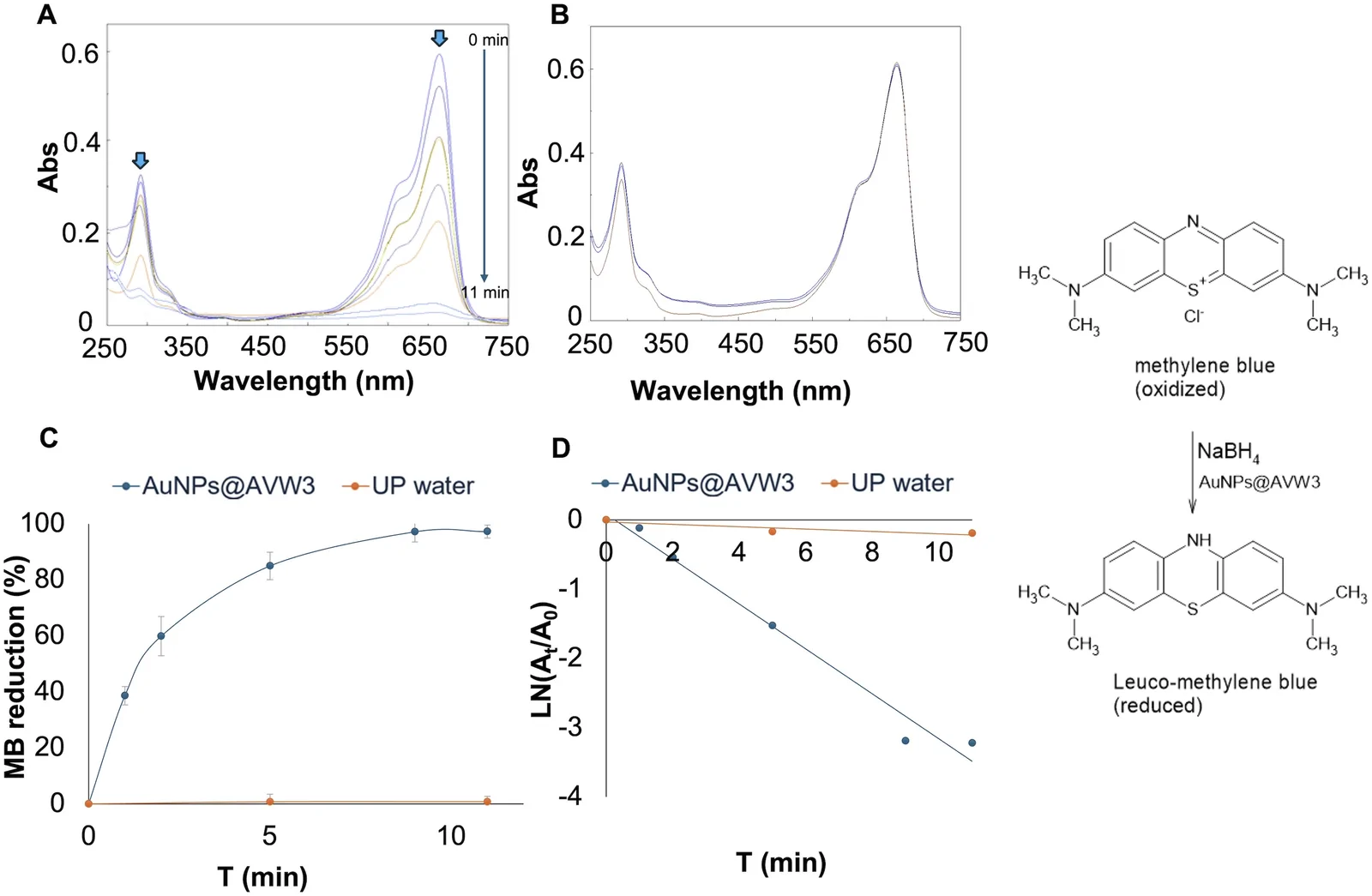
Catalytic reduction of MB in the presence of NaBH4 and AuNPs@AVW3 (A); control MB reduction experiment without AuNPs@AVW3 (B); comparison in the % of reduction of MB in the presence and without AuNPs@AVW3 (C); kinetic studies (D).

**Fig. 7:**
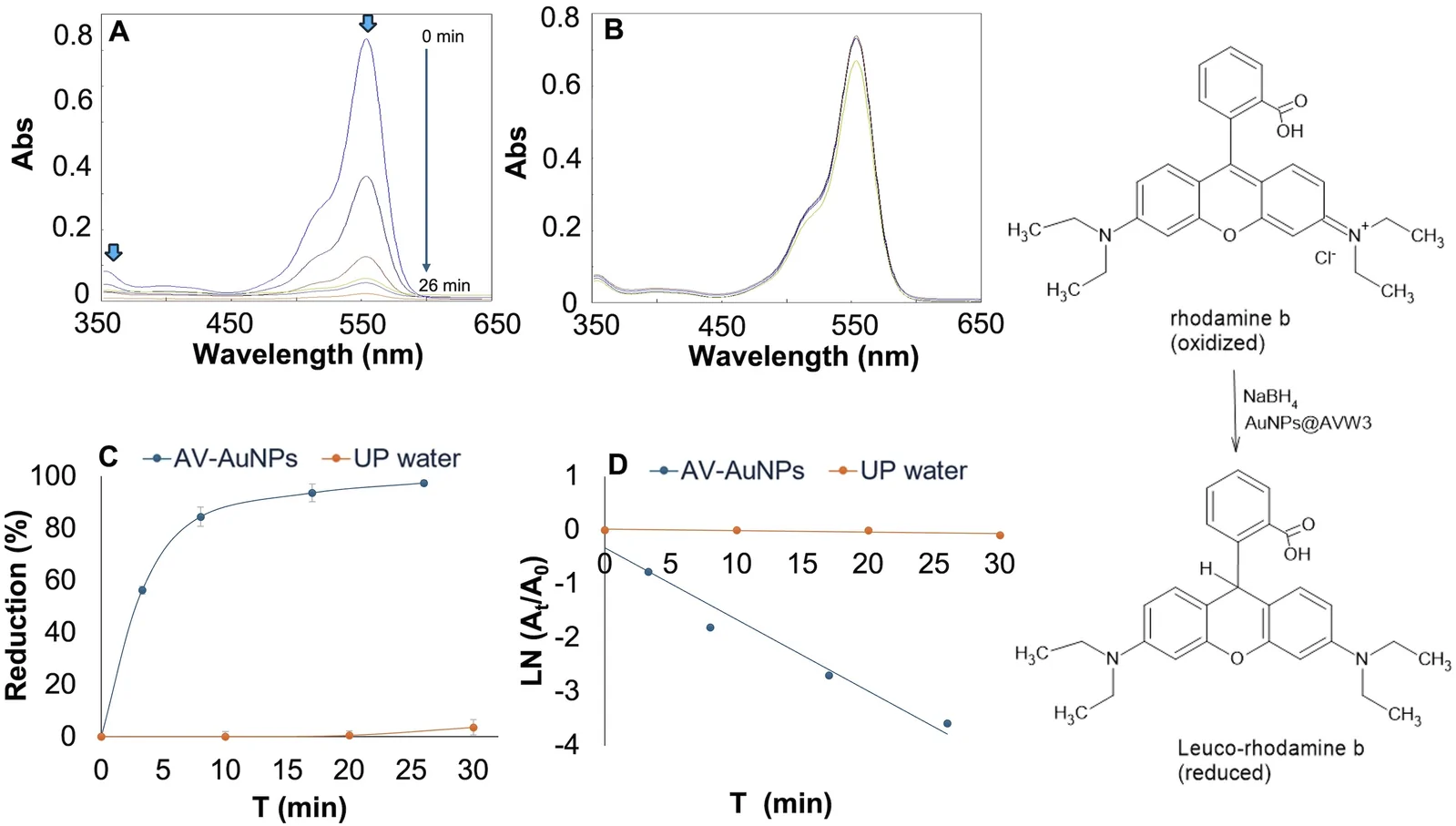
Catalytic reduction of ROb in the presence of NaBH4 and AuNPs@AVW3 (A); control ROb reduction experiment without AuNPs@AVW3 (B); comparison in the % of reduction of ROb in the presence and without AuNPs@AVW3 (C); kinetic studies (D).

**Fig. 8:**
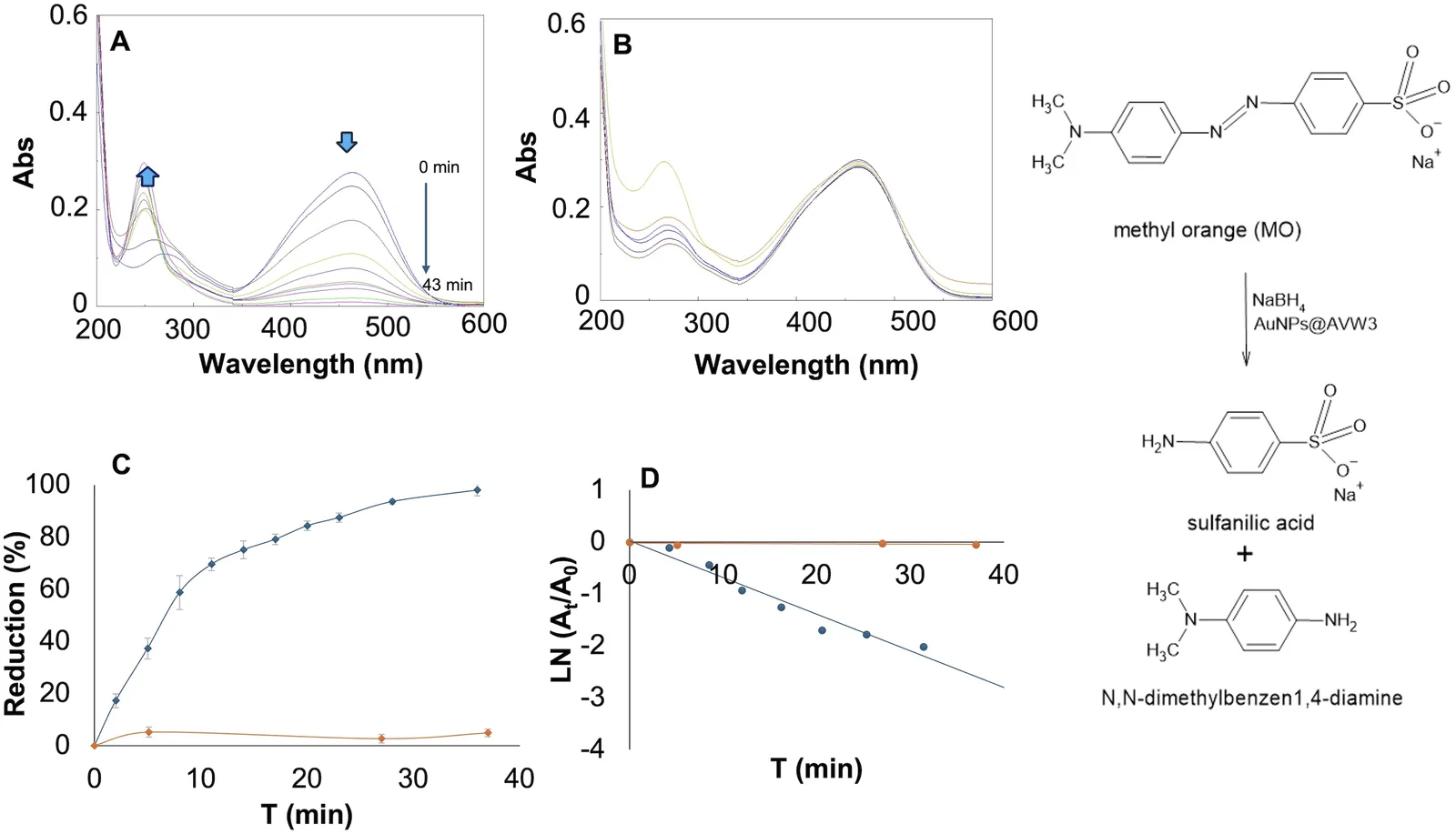
Catalytic reduction of MO in the presence of NaBH4 and AuNPs@AVW3 (A); control MO reduction experiment without AuNPs@AVW3 (B); comparison in the % of reduction of MO in the presence and without AuNPs@AVW3 (C); kinetic studies (D).

**Fig. 9:**
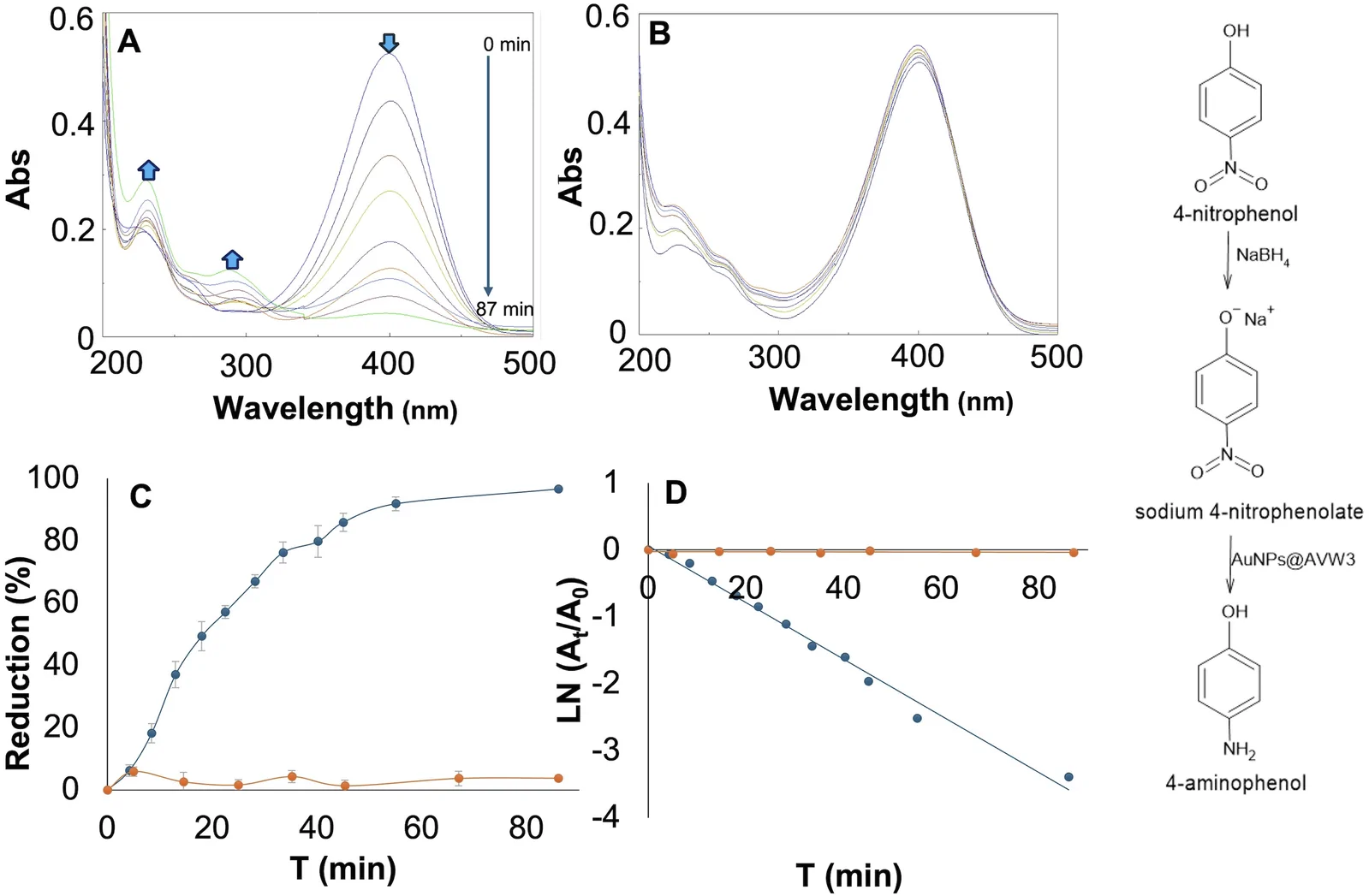
Catalytic reduction of 4NP in the presence of NaBH4 and AuNPs@AVW3 (A); control 4NP reduction experiment without AuNPs@AVW3 (B); comparison in the % of reduction of 4NP in the presence and without AuNPs@AVW3 (C); kinetic studies (D).

Kinetic studies were conducted processing UV-vis absorption data with pseudo first order and pseudo second order equations.

The best results for all pollutants were obtained with the pseudo first order kinetic model with correlation coefficient (*R*^2^) values higher than 0.96. The obtained graphics were shown in Fig. 6D, 7D, 8D and 9D and the constant rate *k* and *R*^2^ were reported in Table 3.

**Table 3:** Comparison of rate constants (*k*), reduction % and times of reduction of each pollutants using AuNPs@AVW3

Pollutant	Abs (nm)	*k* (min^−1^)	*R* ^2^	Reduction %	Reduction time (min)
MO	464	0.09 ± 0.02	0.97	96.5 ± 2.16	43.0 ± 9.9
ROb	554	0.15 ± 0.03	0.96	98.2 ± 1.44	26 ± 3.2
4NP	400	0.04 ± 0.003	0.99	96.8 ± 0.264	87 ± 1.41
MB	664	0.32 ± 0.01	0.98	97.0 ± 1.41	11 ± 5.3

First experiments were focused on cationic dyes such as MB and ROb, synthetic and complex organic pollutants widely used in the textile, paper, and printing industries. MB is classified as a thiazine dye characterized by the presence of a central ring containing sulfur and nitrogen atoms, while ROb represents a member of xanthene dyes with a tricyclic core structure. Comparing reduction performance of AuNPs@AVW3 on cationic dyes (Fig. 6A and 7A), a faster reduction of both MB and ROb in their less toxic leuco forms can be observed, compared to the control experiments conducted without nanocatalyst (Fig. 6B and 7B). A linear correlation between the ln(*A*_*t*_/*A*_0_) and the reaction time confirmed the presence of a pseudo-first-order reaction with rate constant calculated for MB and ROb equal to 0.32 and 0.15 min^−1^ respectively and with a % reduction for MB of 97% in 11 minutes and for ROb of 98.2% in about 19 minutes (see Table 3). Then, the studies were extended on anionic azo-dyes and phenolic compounds using as models MO and 4NP respectively. 4NP possesses acidic properties (p*K*_a_ of approximately 7.15) and in aquatic environments and industrial effluents at neutral or basic pH, it tends to deprotonate, transforming into the negatively charged 4-nitrophenoxide ion (anion). As it can be seen in Fig. 8A and 9A, AuNPs@AVW3 showed also in these cases a catalytic effect compared to control experiments (Fig. 8B and 9B), with a near-complete reduction in 43 minutes (*k* = 0.09 min^−1^) and 87 minutes (*k* = 0.04 min^−1^) for MO and 4NP respectively. Without nanocatalyst, no significant reduction of pollutants was observed (Fig. 8C and 9C). However, their reaction rates were slower than the reaction rates measured for cationic pollutants (see Table 3).

As previously mentioned, all tested pollutants showed a pseudo first order kinetic that is typical of reduction reactions catalysed by nanoparticles and describe a bimolecular reaction between the reducing agent and each pollutant.^11^ The different catalytic behaviour of nanoparticles towards selected pollutants can be justified by analysing the physico-chemical properties of nanoparticles and the chemical nature of pollutants. Reduction reactions mediated by gold nanoparticles, similar to all heterogeneous catalytic processes, proceed through sequential steps located on the interface between the liquid phase and the solid nanocatalyst surface. Firstly, BH_4_^−^ ions and pollutants were adsorbed on AuNPs@AVW3 surface and then electrons were transferred from BH_4_^−^ ions to nanoparticles surface. The gold nanoparticle acts as an electron reservoir that causes a rapid electron transfer to the trapped pollutant, favoring an enhancement of reduction rate. Finally, the reduced products are desorbed, leaving the catalytic sites free. The presence of organic layers around gold nanoparticles contributes to strengthening this effect. Indeed, organic phytochemicals can act as effective electron mediators facilitating the transfer of electrons from reducing agents to hazardous pollutants, further accelerating the reduction process.^40^ In this way, persistent organic pollutants commonly present in industrial wastewater, turn them into less harmful substances. In particular, while MB and ROb are reduced in their leuco forms and 4NP in 4-aminophenol, the azo group(–N=N)– present in MO is degraded to the amine group during the reduction, producing in all cases less toxic substances.^15,41–43^ Since nanocatalyst has an external organic layer negatively charged (as confirmed from FTIR and Zeta Potential analyses), its behavior in the catalytic reduction process can change depending on the nature of the pollutant. As can be seen in Table 3 cationic pollutants showed higher rate constant compared with other studied pollutants. The presence of negative charges around gold nanoparticles causes an electrostatic attraction of cationic pollutants, concentrating them around gold nanoparticles, favoring electron transfer and consequently enhancing reaction rate. Moreover, synthetized gold nanoparticles showed good stability with reduction performance that remains repeatable even after 5 months of storage. Some important studies on catalytic reduction of water pollutants using modified green gold nanoparticles, obtained substituting hazardous compounds with natural extracts, are summarized in the following table and compared with the results obtained in this work (Table 4). As it can be seen, AuNPs@AVW3 require considerably smaller amount of catalyst, compared to other literature works, emphasizing the sustainability of the developed system.

**Table 4:** Comparison of catalytic performance of different green AuNPs in water pollutants reduction; catalyst dosage was calculated in the final volume of reaction

Catalyst	Green extract	Size (nm)	Pollutant	Catalyst dosage (µg mL^−1^)	Reduction time (min)	Reduction %	*k* (min^−1^)	Ref.
Au-CH-CC composite	Chitosan-coated cotton cloth	—	MB	—	12	93	0.18	2
			ROb	—	9	86	0.23	
			Congo red	—	6	98	0.54	
CNS-AuNPs	Catunaregam spinosa extract	25.5	4NP	200	120	96	0.0107	44
ECBP-AuNPs	Empty cotton boll peels extract	22.9	MB	31	8	95.6	0.07	45
			MO	31	9	93.3	0.05	
LC-AuNPs	Litsea cubeba fruit extract	8–18	4NP	1000	10	100	0.35	46
CNP-AuNps	Canthium parviflorum extract	14.1	4NP	2000	24	100	0.09	47
AuNPs@AVW3	*Aloe vera barbadensis* waste extract	10	MB	7	11	97	0.32	This work
			ROb	7	26	98.2	0.15	
			MO	7	43	96.5	0.09	
			4NP	7	87	97	0.04	

Moreover, in order to evaluate the real catalytic action of synthetized nanocatalyst, other control experiments were conducted. Firstly, each pollutant was incubated with AuNPs@AVW3 in the absence of NaBH_4,_ replaced with 1 mL of ultrapure water. In this case, considering the same reduction time reported in Table 2 for each pollutant, only a % reduction less than 6% was obtained for all pollutants. Finally, to evaluate the possible intrinsic reduction capability of the extract, a second set of experiments were performed incubating each pollutant only with the diluted extract (1 : 10; v/v) used in nanoparticles preparation (AVW3). The results showed in all cases non-significant reduction (<2%), confirming the catalytic action of synthetized nanocatalyst.

### Evaluation of simultaneous catalytic reduction of pollutants mixture

A further study was conducted evaluating the ability of green nanocatalyst to reduce all studied water pollutants present simultaneously in solution to simulate a real condition where each pollutant can be concurrently present in complex matrices. To this aim, an aqueous solution containing MB, ROb, MO and 4NP was prepared and the catalytic performances of AuNPs@AVW3 in the presence of NaBH_4_ were evaluated. The reaction was followed by recording Uv-vis spectra at different intervals of time. Generally, in a complex matrix the reduction process of each pollutant can differ from individual analysis. Reaction rates of each pollutant are strongly influenced from the chemical nature and the steric hindrance of compounds that compete for binding to the active sites on nanoparticles surface.

Although in a mixture it is difficult to monitor the wavelengths of each pollutant, at time 0 it is possible to distinguish the characteristic peak of MB (664 nm) that drastically decreases after 1 minute of reaction with a % of reduction of about 87% (Fig. 10). The positive charge and the planar structure of MB maximize the electrostatic attraction favoring a fast adsorption on the gold surface and inducing a fast reduction. However, after 3 hours of reaction all pollutants were almost completely reduced except for ROb, as it can be seen in Fig. 10 with the presence of its typical peak at 550 nm. Although ROb has a positive charge, in presence of other competitors, its complex nonplanar tridimensional structure strongly hinders its access to the active sites present on the nanocatalyst surface causing a drastic decrease of reaction rate compared to the analysis of the single component. The competition between water pollutants favors the reduction of MB, penalizing other pollutants that, because their sterical hindrance (ROb) or their negative surface charge (MO and 4NP) is limited in the interaction with nanocatalyst surface, drastically reducing their reduction rate. However, this experiment demonstrated the potential applicability of AuNPs@AVW3 also in complex matrices since, in about 3 hours, almost all pollutants have been degraded.

**Fig. 10:**
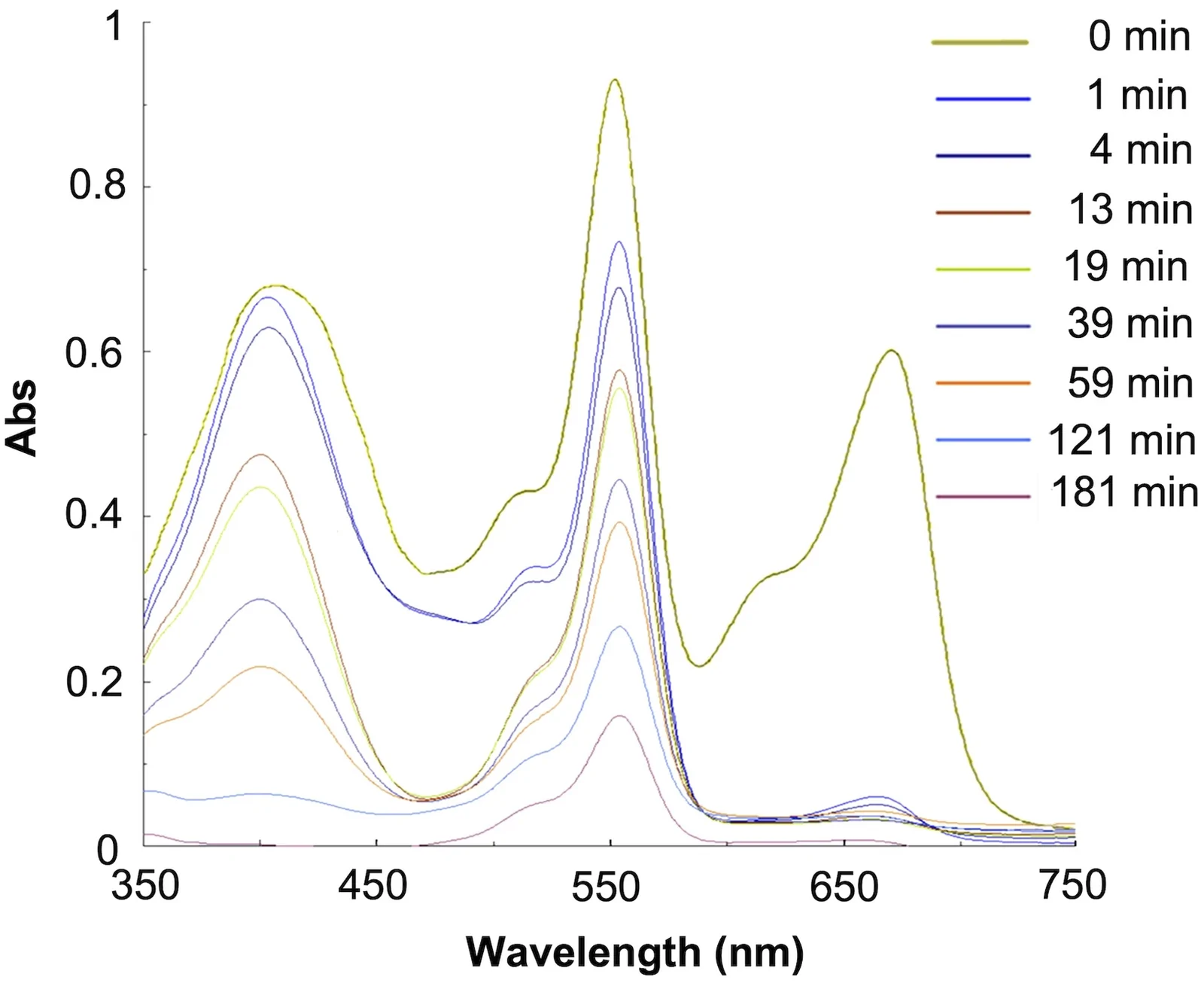
Catalytic power of AuNPs@AVW3 in the simultaneous reduction of water pollutants present in a mixture (MB + MO + ROb + 4NP).

## Conclusion

In this study *Aloe vera* waste extract was successfully used to synthesize biogenic gold nanoparticles through a simple, cost-effective and environmentally friendly alternative to water remediation. Very stable triangular and spherical green AuNPs with a diameter of 10 nm and a ZP of −32.3 mV were obtained. FTIR characterization revealed the presence of an organic layer around synthetized AuNPs composed primarily from sugars (α- and β-glucose and sucrose), polysaccharides (acemannan) and organic acids (mainly malic acid), that contribute to reduce and stabilize the colloidal suspension. Catalytic performance of synthetized green AuNPs were evaluated on cationic and anionic dyes (MB, ROb and MO) and on a phenolic compound (4NP), revealing an excellent catalytic effect especially on cationic dyes with a near-complete reduction in 11 minutes (*k* = 0.32 min^−1^) and 19 minutes (*k* = 0.15 min^−1^) for MB and ROb respectively. Good catalytic performance in the reduction of MO and 4NP was also demonstrated, obtaining a near-complete reduction in 43 minutes (0.09 min^−1^) and 87 minutes (*k* = 0.04 min^−1^) respectively. Finally, the suitability of synthesized nanotool was also demonstrated simulating more complex conditions with the simultaneous presence of each pollutant in a water solution, obtaining a good reduction of all pollutants in approximately 3 hours.

## Author contributions

Lucia Mergola: conceptualization, data curation, formal analysis, investigation, methodology, project administration, supervision, visualization, writing – original draft, and writing – review and editing. Tina L. Capodilupo: conceptualization, data curation, formal analysis, investigation, and writing – review and editing. Antonio Serra; data curation, formal analysis, and writing – review and editing. Daniela Manno: data curation, formal analysis, and writing – review and editing. Alessandro Buccolieri: data curation, formal analysis, and writing – review and editing. Roberta Del Sole: conceptualization, data curation, formal analysis, funding acquisition, project administration, resources, supervision, writing – original draft, and writing – review and editing.

## Conflicts of interest

There are no conflicts to declare.

## Data Availability

The data supporting the findings of this study are available from the corresponding authors upon reasonable request.
